# Growth performance and feed utilisation of Australian hybrid abalone (*Haliotis rubra* × *Haliotis laevigata*) fed increasing dietary protein levels at three water temperatures

**DOI:** 10.1017/S0007114523002519

**Published:** 2024-03-28

**Authors:** Abdul Lathiff Inamul Hassan, Thomas S. Mock, Kieren Searle, Melissa M. Rocker, Giovanni M. Turchini, David S. Francis

**Affiliations:** 1 Nutrition and Seafood Laboratory, School of Life and Environmental Sciences, Deakin University, Geelong, VIC 3225, Australia; 2 School of Agriculture, Food and Ecosystem Sciences (SAFES), The University of Melbourne, Melbourne, VIC, Australia

**Keywords:** Hybrid abalone, *Haliotis rubra*, *Haliotis laevigata*, Protein, Nutrition

## Abstract

Determining the macronutrient requirements for commercially valuable aquaculture species remains crucial for maximising production efficiency. Yet, such information is lacking for Australian hybrid abalone (*Haliotis rubra* × *Haliotis laevigata*), particularly with respect to life stage and water temperatures. The present study aimed to evaluate the effect of dietary protein inclusion level on the growth performance, nutrient utilisation and nutritional quality of juvenile (3·3 g) Australian hybrid abalone reared at three different temperatures representative of winter (12°C), average annual (17°C) and summer (22°C) grow-out periods and fed five diets containing graded dietary protein levels of 35, 38, 41, 44 and 47 %. Abalone growth increased with increasing water temperature with weight gains of approximately 100, 280 and 380 % of their initial weight at 12, 17 and 22°C, respectively. Furthermore, the present study clearly demonstrated that higher dietary protein inclusion levels (41 %) than those currently used commercially (35 %) would significantly improve the growth performance when water temperatures are ≥17°C without any adverse impacts on nutrient utilisation, nutrient deposition or nutritional quality of the abalone soft tissue. For example, at 22°C abalone fed a diet containing 41 % protein obtained a significantly higher weight gain percentage (421 %) compared with those fed a diet containing 35 % protein (356 %). Lastly, it is suggested that maintaining a dietary protein inclusion level of 35 % or implementing a ‘least cost’ feeding approach during cooler seasons, or where water temperatures are ∼12°C, may be beneficial, considering only marginal growth improvements were observed during these periods of slow growth.

Australian abalone production is dominated by wild catch; however, decreasing catches over the last two decades have prompted the abalone aquaculture industry to expand production to cater for a growing consumer demand^(1,2)^. Blacklip abalone (*Haliotis rubra*), greenlip abalone (*Haliotis laevigata*) and their hybrid (*Haliotis rubra × Haliotis laevigata*) are the major cultured species, farmed in slab concrete tanks using a flow-through system with water pumped from the adjacent ocean. During their 3–3·5-year grow-out period, abalone experience seasonal fluctuations in water temperature and the water temperature may vary from 10°C in winter to 25°C summer^(3,4)^. As these water temperature fluctuations often go beyond the optimal thermal tolerance limit for abalone, there is growth retardation leading to an extended culture period, particularly during the cooler months, while sub-optimally high water temperatures can lead to poor health outcomes and mortality events, both ends of this temperature spectrum can ultimately affect the production and profitability of the industry.

Considering that controlling the temperature of large volumes of seawater is currently not feasible in most abalone aquaculture systems, the Australian abalone aquaculture industry has a firm focus on nutritional manipulation as the only feasible option to overcome seasonal impediments to abalone growth performance within well-established existing culture systems and practices (AAGA pers. comm). A series of in-depth nutritional studies, demonstrating the potential to reduce feed cost and improve the growth and health condition of both Australian greenlip and blacklip abalone, have attested to the value of nutritional innovation within the industry^(3–10)^. In this context, a recent study on sub-adult Australian hybrid abalone (*Haliotis rubra × Haliotis laevigata*) at three different rearing temperatures (12, 17 and 22°C) clearly showed that hybrid abalone could achieve better growth performance when fed a higher dietary protein inclusion level, particularly at water temperatures exceeding 17°C, necessitating the need for multi-diet feeding strategies specific to abalone species and water temperature^(11)^.

Abalone are poikilothermic animals and their thermal tolerance levels significantly impact feeding and metabolic activities, ultimately influencing to growth^(12,13)^. However, abalone thermal tolerance levels are both species^(14)^ and size^(15,16)^ specific. Further, the experimental findings relating to the optimal size-specific protein requirement for abalone appear species-specific. Stone *et al.* ^(4)^ suggested a higher optimal dietary protein requirement for smaller size abalone, suggesting 29 and 24 % dietary protein for 1-year-old (3–6 g) and 2-year-old greenlip abalone (28–34 g), respectively. In contrast, nutritional studies with *Haliotis midae* revealed higher optimal protein requirements (44 %) for larger size abalone (7–14 g) compared with smaller size abalone (0·2–1 g, 34 % dietary protein level)^(17)^. However, no such studies concerning Australian hybrid abalone have been undertaken; therefore, it is unclear whether the Australian hybrid abalone also has size-specific protein requirements similar to the trends observed in other abalone species. Further, the recent finding on sub-adult Australian hybrid abalone related to better growth at higher temperature when fed on higher protein diet by the same authors^(11)^ has prompted them to investigate whether the same growth potential may be attained in juvenile Australian hybrid abalone.

Feeding strategies, including feed formulations adapted to both animal size and water temperature, are widespread in well-developed finfish and crustacean aquaculture^(18)^. Recent investigations in emerging species, such as greenlip abalone, have demonstrated that the optimisation of dietary protein levels specific to culture temperature and abalone size has resulted in improved growth performance, reduced culture duration and better economic returns^(4,19)^. Considering the efficiencies seen in greenlip abalone culture and other aquaculture species, the Australian hybrid abalone aquaculture industry has a pressing need to develop a temperature and size-specific feeds to fully realise the potential of this industry.

In this context, the current study aimed to establish the optimal protein requirements of farmed juvenile Australian hybrid abalone with respect to three water temperatures, representative of winter, optimal and summer grow-out periods. Given the identified gaps in reported knowledge with respect to hybrid abalone, a significant contribution towards the development of life stage and seasonally specific feeds for hybrid abalone to facilitate the projected growth of the Australian abalone aquaculture industry is expected.

## Materials and methods

### Experimental system, animals and stocking

The experiment was conducted using a flow-through seawater system in air temperature (20°C) and photoperiod controlled (12 h complete dark and 12 h low-intensity light) facility at Deakin University, Queenscliff Marine Science Centre, Queenscliff, Victoria where the abalone were fed the experimental diets for a period of 150 d. Three water temperatures representative of winter (12°C), summer (22°C) and the annual average water temperature (17°C), respectively, typical of abalone aquaculture farms in Victoria, Australia. There were fifteen culture units (12·5 l blue plastic rectangular tanks, dimensions of 39·2 cm × 28·8 cm × 11·0 cm) at each experimental temperature. Temperature-controlled, UV treated and filtered (5 µm and 1 µm cartridge), sea water supplied at a flow rate of 400 ml/min. Water depth was maintained at 8·5 cm to give an effective water volume of 9·6 l, and the water was aerated using air stone to keep dissolved oxygen level near saturation. A hide, made from celuka board and ceramic tiles, was placed in each culture unit to increase the effective surface area for attachment. In addition, a 2-cm wide synthetic grass turf strip was fastened around the inner perimeter of the tank, above the water line to prevent abalone escapees.

One-year-old Australian hybrid abalone were obtained from Jade tiger abalone farm (Craig Mostyn Group) in September 2019. Abalone were anaesthetised and transported to the experimental facility within 30 min of collection and, upon arrival, held temporarily in two well-aerated flow-through seawater tanks with a set water temperature representative of on-farm conditions. Abalone were acclimated to the experimental system for 2 weeks prior to feeding the experimental diets and fed a restricted ration of an acclimation diet made using the same ingredients as the experimental diets and formulated to contain 30 % protein. This was achieved by feeding the abalone a ration that was below the expected specific feeding rate for abalone of this size, as informed by the supplier and previous research^(20)^. Furthermore, during this period, daily monitoring ensured that no feed was remaining in each tank the following day after feeding. Feeding was restricted to minimise potential size variation between dietary treatments prior to the experimental period. During the acclimation period, the water temperature was altered by 1°C/d until the nominated experimental temperature was reached. Water temperatures were held within ±1°C of the nominated experimental temperature throughout the growth trial by using 5 horsepower heat pump temperature control units with 24 and 16 kW of heat and chilling output, respectively (Aquahort Ltd.). At the beginning of the experiment, thirty abalone were weighed and shell-length measured and assigned to each of the culture units while minimising variability among individual weights and total biomass. Each culture unit was randomly assigned to one of five experimental dietary treatments within each of the three experimental temperatures, meaning there were fifteen culture units for each experimental temperature and 1350 individual abalone across forty-five culture units in total.

### Experimental diets and feeding

Five experimental diets were formulated to contain graded dietary protein levels: 350 g/kg (P35), 380 g/kg (P38), 410 g/kg (P41), 440 g/kg (P44) and 470 g/kg (P47) by increasing the inclusion of major protein sources, namely soya protein isolate, casein and lupin meal and decreasing the levels of pregelatinised wheat starch (Table 1). All other dietary ingredients were identical and included at similar levels across all the experimental diets. Fish oil and rapeseed oil were used as the predominant lipid source, and dietary lipid levels were formulated to be relatively low (3–4 %) in line with current commercial formulations considering the poor lipid digestive capacity of abalone. Diets were formulated to be isoenergetic (17–18 MJ/kg). The amino acid composition of the experimental diets was balanced to match the soft tissue composition of parent abalone species (*Haliotis laevigata* and *Haliotis rubra*) due to a lack of amino acid composition data on Australian hybrid abalone and was also informed by published nutritional profiles for diets fed to the abalone parent species in previous research. Specifically, the nutritional profiles of both abalone tissue and diets were identified from published literature using a combination of key word and ad hoc search techniques using Web of Knowledge® and Google Scholar® databases. Data were only used if complete, or near complete, amino acid profiles were reported. Specifically, amino acid data of both wild and farmed abalone from Fleming *et al.* ^(7)^, Coote *et al.* ^(21)^ and Daume *et al.* ^(22)^ were extracted and used to inform subsequent dietary formulations. Yttrium oxide was added to the diets at an inclusion rate of 0·5 % for subsequent determination of digestibility coefficients (data not presented herein). All the dietary ingredients were analysed for proximate composition before diet formulation (data not reported). Experimental diets were cold-pressed into flat pellets (diameter ∼ 4 mm) using a commercial bench-top pellet press. Briefly, the dry ingredients were mixed thoroughly using a Hobart 20 l planetary mixer (Hobart) prior to the addition of oils. Subsequently, hot water was added until the ‘mash’ formed a consistent bolus, which typically required the addition of 250 g of water per kg of mash. After pressing, the pellets were dried at 35°C for 48 h in a dryer with air extraction. Abalone were fed their respective diet to satiation daily between 1600 and 1700 h to ensure growth was not limited by diet availability. Feed consumption was recorded by counting the number of uneaten pellets and converting to an ‘as fed’ mass using the average weight of a pellet determined prior and subsequently subtracting the uneaten mass from the total mass fed. The feeding rate was adjusted by increasing the daily feed in each tank by 0·5 g/d when the number of uneaten pellets fell below 20/tank. Culture units were cleaned daily between 0800 and 1000 h by syphoning out uneaten feed and faeces.

**Table 1.** Ingredient composition of experimental diets (g/kg)

	Experimental diets*				
	P35	P38	P41	P44	P47
Soya protein isolate	126·3	144·4	162·4	180·5	198·6
Lupin meal	63·2	72·2	81·2	90·3	99·3
Casein	126·3	144·4	162·4	180·5	198·6
Pregelatinised wheat starch	429·1	385·9	342·7	299·4	256·2
Diatomaceous earth	34·3	33·0	31·8	30·5	29·2
Fishmeal	50·0	50·0	50·0	50·0	50·0
Rapeseed oil	25·3	24·6	24·0	23·3	22·7
Gluten	50·0	50·0	50·0	50·0	50·0
Gelatin	50·0	50·0	50·0	50·0	50·0
Lecithin	3·0	3·0	3·0	3·0	3·0
Agar	5·0	5·0	5·0	5·0	5·0
Sodium alginate	5·0	5·0	5·0	5·0	5·0
Yttrium oxide	0·5	0·5	0·5	0·5	0·5
Vitamin and mineral mix	4·0	4·0	4·0	4·0	4·0
Vitamin C	0·5	0·5	0·5	0·5	0·5
Choline	5·0	5·0	5·0	5·0	5·0
Monosodium phosphate	7·5	7·5	7·5	7·5	7·5
Methionine	6·0	6·0	6·0	6·0	6·0
Lysine	6·0	6·0	6·0	6·0	6·0
Arginine	6·0	6·0	6·0	6·0	6·0
Threonine	6·0	6·0	6·0	6·0	6·0
Calcium sulphate	5·0	5·0	5·0	5·0	5·0
Vitamin E	1·0	1·0	1·0	1·0	1·0

* Experimental diets: P35 = 350 g/kg protein, P38 = 380 g/kg protein, P41 = 410 g/kg protein, P44 = 440 g/kg protein, P47 = 470 g/kg protein.

### Water quality management

Water temperature and dissolved oxygen were monitored daily using a handheld dissolved oxygen metre. Salinity and pH were measured weekly using a refractometer (Atago® S/Mill hand refractometer) and pH metre (Apera Instruments® PH20 pH tester), respectively. Flow rates were checked weekly using a flowmeter and held at 500 ml/min throughout the growth trial. Cartridge filters (5 and 1 µm) were cleaned weekly to ensure adequate water flow.

### Growth performance

Abalone were weighed on a wet weight basis after blot drying the excess water using a cloth towel. Shell lengths were measured across the longest axis using vernier callipers. All feed weight measurements were made on as-fed basis. The feed consumption, biometry and growth performance indices such as specific growth rate (SGR), shell growth rate, feed conversion ratio (FCR), protein efficiency ratio, energy efficiency ratio, protein deposition, energy deposition and condition factor were calculated as described in detail by Britz *et al.* and Bansemer *et al*. Dead abalone weights were accounted for when calculating biomass gain and FCR estimation. The formulae used to calculate the parameters above were as follows:

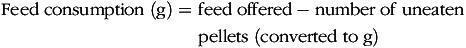




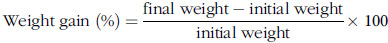




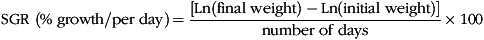




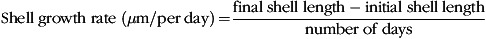




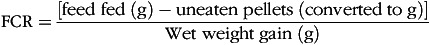




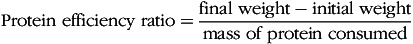




Corresponding formula was used to calculate the energy efficiency ratio.

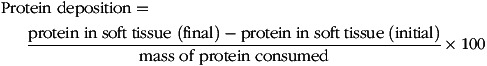




Corresponding formula was used to calculate the energy deposition.

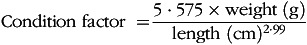




### Biochemical analyses

At the beginning of the experiment, an initial sample of 20 abalone was taken and stored immediately at −20°C for subsequent biometry and chemical analysis. Similarly, at the end of the trial, after 150 d of feeding the experimental diets, seven abalone were collected from each culture unit and immediately stored at −20°C until subsequent analysis. Moisture, ash, crude protein and crude lipid contents of the dietary ingredients, experimental diets and abalone soft tissue were determined using oven drying at 80°C to a constant weight, incinerating in a muffle furnace at 550°C, automated Kjeltec 2300 (Nitrogen conversion factor of 6·25) and dichloromethane: methanol (2:1) cold extraction of Folch *et al.* ^(23)^, respectively, as reported in detail by Mock *et al.* ^(24)^. Nitrogen-free extract was calculated by subtracting crude protein, crude lipid and ash from 100 %.

The amino acid composition of the experimental diets and abalone soft tissue was identified and quantified using a reverse-phase HPLC (1260 Agilent infinity II series systems, Agilent Technologies) by derivatising the acid hydrolysed sample (using 6 M HCl) with o-phthaldialdehyde and fluorenylmethyloxycarbonyl chloride as described in detail by Lewis *et al*.^(25)^

### Statistical analyses

All the data, except ingredient and proximate composition of experimental feeds, were reported as mean values with their standard error of mean, and replicate data were pooled for each treatment (*n* 3). Upon confirmation of homogeneity of variance and normality using Levene’s test and Shapiro–Wilk test, respectively, data were subjected to a two-way ANOVA. Where there was a significant interaction between the two independent factors (dietary protein level, *n* 5 and water temperature, *n* 3), one-way ANOVA with Tukey’s post hoc test of multiple comparisons was performed for the response variable across all treatment groups (*n* 15). Where no significant interaction was recorded, one-way ANOVA and Tukey’s post hoc test of multiple comparisons were performed between dietary protein levels within each experimental temperature separately. Regression analyses (second-order polynomial regression) were performed separately at each temperature against dietary protein level for key performance parameters. Power analysis was conducted prior to the commencement of the experiment according to methods outlined in Cohen *et al*.^(26)^ This was conducted in conjunction with logistical considerations, such as the experimental system constraints and the optimal stocking density for ‘normal’ growth and behaviour to inform the number of replicates and the number of experimental animals within each replicate. Hence, three replicate units were assigned to each treatment and thirty abalone were assigned to each replicate unit (tank). Given the above, power analysis, where significance was accepted at *P* ≤ 0·05 and statistical power set to 80 %, revealed an effect size of 0·33. This determined effect size, designated arbitrarily as between small and large, by Cohen *et al.* ^(26)^ was considered acceptable in order to observe expected biological effects due to the experimental treatments. For all statistical analysis, significance was accepted at *P* ≤ 0·05 and all analyses were performed using R (Version 3.6.3, R Core Team 2020).

## Results

### General observations

The experimental feeds were formulated to be isoenergetic and isolipidic, which were confirmed by proximate analysis (Table 2). As expected, the dietary protein levels and amino acid concentrations gradually increased between P35 and P47. There was no significant difference in initial weight (3·27 (sem 0·02) g) and initial shell length (28·1 (sem 0·06) mm) across the experimental treatments or between water temperatures. Abalone readily accepted the experimental feeds with no mortalities. The average water temperature and dissolved oxygen concentrations are reported in Table 3, and they remained close to the experimental temperature throughout the duration of the culture.

**Table 2.** Proximate and amino acid composition (mg/g diet as fed) of the five experimental diets fed to juvenile hybrid abalone

	P35*	P38	P41	P44	P47
Proximate composition					
Moisture	45·82	43·73	46·57	36·8	40·58
Protein	350·07	372·15	401·75	436·61	464·61
Lipid	32·48	36·98	30·37	34·07	32·09
Ash	55·79	56·56	55·34	57·17	57·62
NFE†	515·83	490·57	465·96	435·35	405·10
Gross energy (MJ/kg)‡	18·42	18·68	18·70	19·14	19·20
Amino acids					
Aspartic acid	26·1	29·0	32·1	35·4	39·6
Glutamic acid	67·1	72·6	80·1	87·5	96·6
Serine	13·7	13·9	16·9	19·3	21·9
Histidine	7·1	7·8	8·7	9·4	10·4
Glycine	19·5	20·4	21·1	22·5	23·9
Threonine	15·5	16·2	18·1	20·2	21·9
Arginine	21·8	23·6	25·6	27·3	30·2
Alanine	13·8	14·8	15·8	17·3	18·8
Tyrosine	8·0	8·8	11·0	10·9	13·3
Valine	15·9	17·6	18·9	21·0	22·8
Methionine	10·0	10·6	11·6	11·9	12·7
Phenylalanine	13·7	15·0	16·7	18·4	20·5
Isoleucine	14·0	15·7	16·8	18·9	20·7
Leucine	23·5	25·5	28·5	31·4	35·0
Lysine	22·9	23·1	25·5	23·6	29·4
Proline	31·5	32·8	35·5	38·5	42·9
Total amino acids	324·1	347·4	383·0	413·6	460·7

* See Table 1 for detailed experimental feed information.

† Nitrogen-free extract (NFE) = (100- (crude protein + crude lipid + ash)).

‡ Energy was calculated using the values of 17.2, 23.6 and 39.5 MJ/kg for NFE, protein and lipid.

**Table 3.** Water temperature and dissolved oxygen concentrations recorded throughout the abalone growth experiments (Mean values with their standard error of the means)

	12°C		17°C		22°C	
	Mean	SEM	Mean	SEM	Mean	SEM
Temperature (°C)	12·2	0·0	17·1	0·0	22	0·1
Dissolved oxygen (mg/l)	9·7	0·0	8·1	0·6	6·8	0·0
Dissolved oxygen (% saturation)	96·2	0·2	94·6	0·6	93·8	0·2
pH	8·2	0·0	8·2	0·0	8·2	0·0
Salinity (ppt)	35	0·0	35	0·0	35	0·0

### Abalone growth performance

**Fig. 1.**
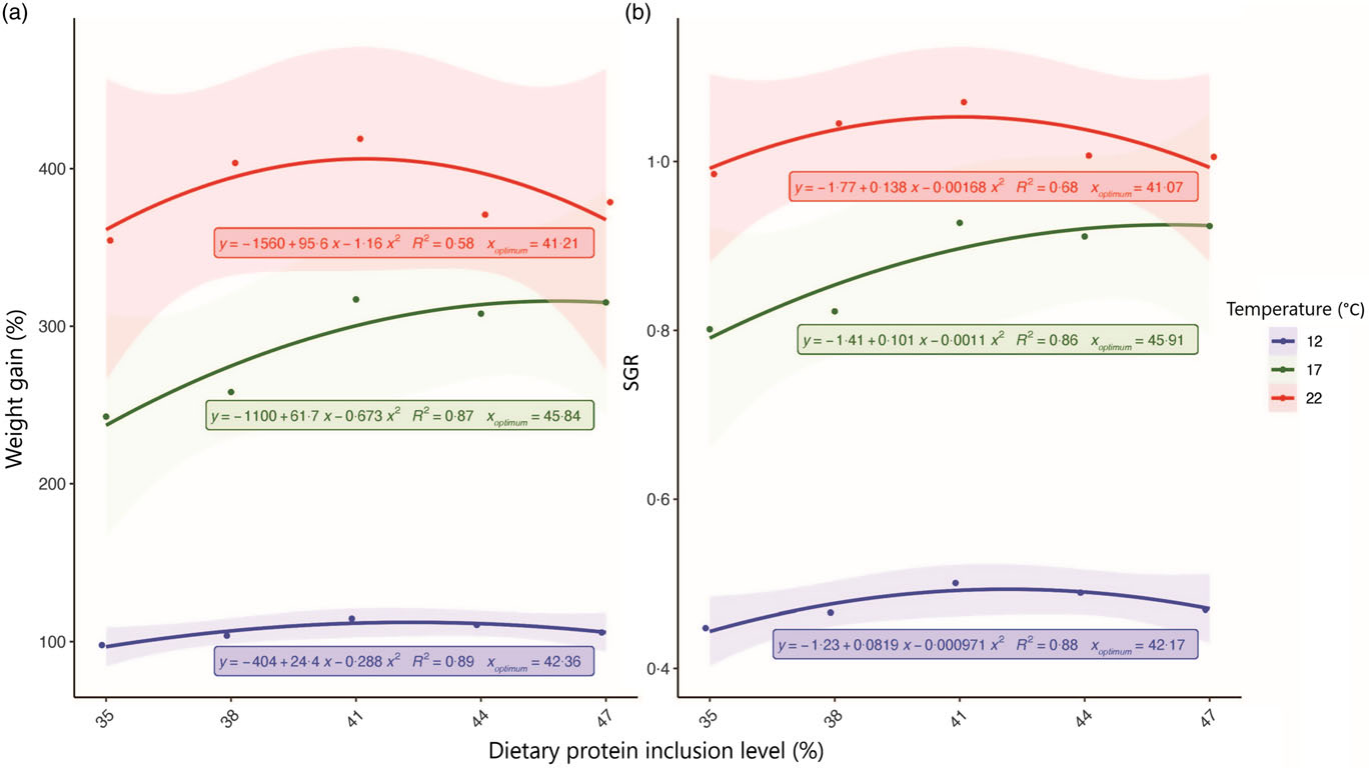
Second-order polynomial regression between weight gain percentage and SGR (weight) (Y-axis) and dietary protein level (X-axis) from juvenile Australian hybrid abalone fed five dietary protein levels at three water temperatures. SGR, specific growth rate.

Both water temperature and dietary protein level significantly affected growth performance parameters, and an interactive effect of protein and temperature was recorded for feed consumption. There was a clear trend of increased growth performance, including weight gain percentage, shell growth rate, and SGR, as water temperature increased from 12°C to 22°C and dietary protein level increased from P35 to P41 (*P* < 0·05), and growth performance did not improve beyond P41 (Table 4 and Fig. 1). While FCR was also significantly affected by both water temperature and dietary protein level, it was lowest (1·00–1·26) at 17°C. While feed consumption improved with water temperature, it was highest among the higher dietary protein levels, namely P44 and P47. Overall, significant differences in abalone growth performance between dietary treatments mostly observed at the higher water temperatures of 17 and 22°C.

**Table 4.** Growth performance of juvenile Australian hybrid abalone fed diets containing different dietary protein levels at three water temperatures (Mean values with their standard error of the means)

	12°C										17°C									
	P35*		P38		P41		P44		P47		P35		P38		P41		P44		P47	
	Mean	SEM	Mean	SEM	Mean	SEM	Mean	SEM	Mean	SEM	Mean	SEM	Mean	SEM	Mean	SEM	Mean	SEM	Mean	SEM
Initial weight (g)	3·28	0	3·28	0	3·28	0	3·28	0	3·28	0	3·25	0	3·25	0	3·25	0	3·25	0	3·25	0
Initial shell length (mm)	28·1	0·06	28·1	0·06	28·1	0·06	28·1	0·06	28·1	0·06	28·1	0·06	28·1	0·06	28·1	0·06	28·1	0·06	28·1	0·06
Final weight (g)	6·49	0·13	6·69	0·14	7·04	0·14	6·91	0·14	6·75	0·16	11·13	0·28^a^	11·64	0·33^a^	13·55	0·36^b^	13·26	0·37^b^	13·49	0·39^b^
Final shell length (mm)	35	0·24^a^	35·4	0·24^a,b^	36·2	0·27^b^	36·1	0·27^b^	35·8	0·30^a,b^	41·3	0·30^a^	41·7	0·39^a^	43·9	0·42^b^	43·6	0·41^b^	43·5	0·38^b^
Condition factor	0·87	0·01	0·86	0·01	0·85	0·01	0·85	0·01	0·84	0·01	0·9	0·01	0·91	0·01	0·91	0·01	0·91	0·01	0·94	0·02
SGR	0·45	0·01	0·47	0·01	0·5	0·01	0·49	0·01	0·47	0·02	0·8	0·02^a^	0·82	0·02^a^	0·93	0·02^b^	0·91	0·02^b^	0·92	0·02^b^
Survival (%)	100	0	100	0	100	0	100	0	100	0	100	0	100	0	100	0	100	0	100	0
Weight gain (%)	99·1	3·9	105·3	4·4	115·9	4·4	112	4·3	107·1	4·9	241·6	8·5^a^	257·1	10·2^a^	315·8	11·1^b^	306·7	11·4^b^	313·8	12·0^b^
FCR	2·12	0·07	1·9	0·24	1·87	0·1	1·88	0·02	2·1	0·09	1·26	0·09^b^	1·14	0·03^a,b^	1	0·03^a^	1·19	0·05^a,b^	1·13	0·05^a,b^
Shell growth rate (µm/d)	46	1·6^a^	48·6	1·6^a,b^	54·2	1·8^b^	53·1	1·8^b^	51·4	2·0^a,b^	87·8	2·0^a^	90·7	2·6^a^	105·1	2·8^b^	103·6	2·7^b^	102·6	2·5^b^
Feed consumption (g/animal)	6·88	0·04^e^	6·38	0·77^e^	6·98	0·11^e^	6·82	0·04^e^	7·27	0·04^e^	9·95	0·93^d^	9·55	0·17^d^	10·29	0·2^d^	11·9	0·03^c^	11·55	0·09^c^

22°C												
P35		P38		P41		P44		P47				
Mean	SEM	Mean	SEM	Mean	SEM	Mean	SEM	Mean	SEM	Protein (A)	Temperature (B)	A × B
3·27	0	3·27	0	3·27	0	3·27	0	3·27	0	–	–	–
28·1	0·06	28·1	0·06	28·1	0·06	28·1	0·06	28·1	0·06	–	–	–
14·86	0·42^a^	16·37	0·51^a,b^	16·99	0·53^b^	15·4	0·47^a,b^	15·65	0·59^a,b^	0·009	*P* < 0·001	0·16
45·3	0·43	46·8	0·46	47	0·47	45·5	0·44	45·5	0·51	0·004	*P* < 0·001	0·11
0·91	0·01	0·9	0·01	0·92	0·01	0·92	0·01	0·93	0·02	0·75	*P* < 0·001	0·44
0·99	0·02^a^	1·05	0·02^a,b^	1·07	0·02^b^	1·01	0·01^a,b^	1·01	0·01^a,b^	0·002	*P* < 0·001	0·13
100	0	100	0	100	0	100	0	100	0	–	–	–
355·8	12·9^a^	402·1	15·6^a,b^	421·3	16·3^b^	372·3	14·3^a,b^	380·2	18·1^a,b^	0·009	*P* < 0·001	0·15
1·44	0·03	1·23	0·1	1·22	0·13	1·5	0·04	1·45	0·13	0·02	*P* < 0·001	0·69
114·5	2·8	125	3·1	126	3·1	116·2	2·9	116·1	3·4	0·004	*P* < 0·001	0·11
16·62	0·14^b^	16·06	0·28^b^	16·34	0·51^b^	18·22	0·11^a^	17·73	0·22^a^			*P* < 0·001

* See Table 1 for detailed experimental feed information.

Values in the same row with different superscripts are significantly different (two-way ANOVA and Tukey’s post hoc test); where there was no significant interaction between the two independent factors, comparisons between protein levels within temperature have been completed (one-way ANOVA and Tukey’s post hoc test).

At 12°C, the weight gain percentage and SGR ranged from 99·1 % to 115·9 % and 0·45 to 0·50, respectively (Fig. 1 and Table 4). However, dietary protein level only significantly impacted final shell length, shell growth rate and feed consumption, which were lower in P35 compared with other treatments. Similarly, feed consumption was significantly lower at the P38 treatment compared with other treatments.

At 17°C, weight gain percentage ranged from 241·6 % to 315·8 % in treatment P35 and P41, respectively, with corresponding SGR of 0·80–0·93 (Fig. 1 and Table 4). At this temperature, the dietary protein level significantly affected final weight, final shell length, SGR, weight gain percentage, FCR and shell growth rate, where these parameters were, generally speaking, superior with increasing dietary protein levels up to P41. However, growth performance did not significantly increase beyond the P41 treatment. Feed consumption was also significantly impacted by dietary protein level at 17°C and was highest in abalone fed diets containing the highest dietary protein levels tested (P44 and P47).

At 22°C, abalone gained 355·8–421·3 % of initial body weight with corresponding SGR ranging from 0·99 to 1·07 in P35 and P41, respectively (Fig. 1 and Table 4). At this temperature, dietary protein level significantly impacted final weight, final shell length, SGR, weight gain percentage and shell growth rate. All these growth performance indices followed a similar trend to those observed at 17°C, with overall improved growth performance recorded in abalone in the P41 treatment. Similar to 17°C, feed consumption was highest in abalone fed the high dietary protein treatments (P44 and P47).

### Nutrient retention efficiency

Both water temperature and dietary protein level significantly affected nutrient retention parameters. Regardless of dietary treatment, dietary protein and energy deposition and retention appeared to be highest in abalone grown at 17°C and poorest in those grown at 12°C (Table 5). However, differences in nutrient retention efficiency between dietary treatments within temperature were mostly manifest at the higher water temperatures (17 and 22°C). Dietary protein level significantly affected apparent protein efficiency ratio at both 17 and 22°C, and it was significantly higher at P38 and P41, and P38, respectively (Table 5).

**Table 5.** Nutrient retention efficiency of juvenile Australian hybrid abalone fed five dietary protein levels at three water temperature (Mean values with their standard error of the means)

	12°C										17°C									
	P35*		P38		P41		P44		P47		P35		P38		P41		P44		P47	
	Mean	SEM	Mean	SEM	Mean	SEM	Mean	SEM	Mean	SEM	Mean	SEM	Mean	SEM	Mean	SEM	Mean	SEM	Mean	SEM
PER	1·32	0·05^a,b^	1·46	0·18^a^	1·32	0·07^a,b^	1·2	0·01^a,b^	1·01	0·04^b^	2·25	0·16^a,b^	2·37	0·06^b^	2·46	0·06^a^	1·89	0·08^b^	1·88	0·08^b^
PD %	18·01	1·4	21·43	3·7	19·28	1·5	17·4	1·6	16·12	0·2	32·17	3	35·84	1·3	37·3	1·2	32·48	1·4	33·89	1·4
EER	25·3	0·9	29·26	3·7	28·58	1·4	27·76	0·3	24·76	1·1	43·33	3·1^b^	47·36	1·2^a,b^	53·15	1·4^a^	43·85	1·9^a,b^	45·96	1·9^a,b^
ED %	14·62	0·8	17·12	1·5	15·53	1·2	14·57	1·1	12·92	0·4	24·65	2	26·55	0·6	28·89	0·6	25·69	1·4	26	1·2

22°C												
P35		P38		P41		P44		P47				
Mean	SEM	Mean	SEM	Mean	SEM	Mean	SEM	Mean	SEM	Protein (A)	Temperature (B)	A x B
1·97	0·04^a,b^	2·22	0·18^a^	2·07	0·22^a,b^	1·5	0·04^b^	1·48	0·13^b^	*P* < 0·001	*P* < 0·001	0·47
30	1·6^a,b^	34·59	1·3^a^	34·09	3·5^a,b^	24·83	0·9^b^	26·6	2·1a^b^	0·002	*P* < 0·001	0·55
37·83	0·8	44·32	3·6	44·65	4·8	34·68	0·8	36·24	3·2	0·002	*P* < 0·001	0·5
21·91	0·9	25·66	1·9	26·1	3	19·73	0·6	21·33	1·9	0·01	*P* < 0·001	0·64

PER, protein efficiency ratio; PD, protein deposition; EER, energy efficiency ratio; ED, energy deposition.

* See Table 1 for detailed experimental feed information.

Values in the same row with different superscripts are significantly different (two-way ANOVA and Tukey’s post hoc test); where there was no significant interaction between the two independent factors, comparisons between protein levels within temperature have been completed (one-way ANOVA and Tukey’s post hoc test).

### Abalone soft tissue proximate composition

Within the proximate composition of the soft tissue of abalone, there were numerous interactive effects of water temperature and dietary protein level, specifically, protein, lipid, ash and energy (Table 6). For moisture and total energy, a significant effect of both water temperature and dietary protein level was recorded. While lipid was revealed as a minor component of the soft tissue composition of abalone, it was significantly higher in P38, P41 and P47 at 22°C (20·5–21·2 mg/g) compared with P47 at 12°C (16·6 mg/g). Soft tissue protein content increased in-line with dietary protein level, where, regardless of the experimental water temperature, values were highest in P47. However, tissue protein content was numerically higher in the P47 treatment at both 17 and 22°C (175 mg/g) compared with 12°C (161·5 mg/g). Correspondingly, soft tissue concentrations of nitrogen-free extract were highest in P35. Accordingly, soft tissue energy levels were higher in the high dietary protein treatments. While there was relatively difference in total ash content in the soft tissue of abalone, there were slight, yet significant, differences between P38 and P44 at 17°C.

**Table 6.** Proximate and amino acid composition (mg/g soft tissue) of juvenile Australian hybrid abalone fed five dietary protein levels at three water temperatures (Mean values with their standard error of the means)

	12°C										17°C									
	P35*		P38		P41		P44		P47		P35		P38		P41		P44		P47	
	Mean	SEM	Mean	SEM	Mean	SEM	Mean	SEM	Mean	SEM	Mean	SEM	Mean	SEM	Mean	SEM	Mean	SEM	Mean	SEM
Proximate composition (mg/g soft tissue)																				
Moisture	750·6	3·4	751·1	5·7	758·3	5·6	762·7	11	763·7	2	743·6	3	745·6	7	752	0·7	743·3	5	749·1	4·9
Protein	153·2	3·4^c,d^	156·2	4·2^b,c,d^	155·6	4·3^b,c,d^	155·6	8·7^b,c,d^	161·5	4·1^a,b,c,d^	150	6·3^d^	155·3	3·2^b,c,d^	155	4·1^b,c,d^	169	0·9^a,b^	175	2·8^a^
Lipid	18·6	1·6^a,b^	19·3	1·2^a,b^	18·6	1·4^a,b^	18·2	1·3^a,b^	16·6	0·5^b^	18·9	0·3^a,b^	18·7	1·2^a,b^	18·8	0·9^a,b^	19·7	0·9^a,b^	18	0·7^ab^
Ash	20·9	0·5^a,b^	20·2	0·8^a,b,c^	20·7	0·3^a,b,c^	20·3	0·5^a,b,c^	20·4	0·3^a,b,c^	19·6	0·2^b,c^	21·6	0·5^a^	20·7	0·7^a,b,c^	18·9	0·3^c^	21	1·3^ab^
NFE†	56·7	3·5^a^	53·2	2·3^a,b^	46·8	1·8^b,c^	43·2	0·5^c,d^	37·9	2·9^d^	68	4·7^a^	58·8	4·5^a,b^	53·6	5·1^b^	49·2	4·4^b^	37	2·7^c^
Energy (MJ/kg)	53·3	1·0^a,b,c^	53·6	1·5^a,b,c^	52·1	1·3^bc^	51·3	2·7^c^	51·2	0·5^c^	54·5	0·9^a,b,c^	54·2	1·6^a,b,c^	53·2	0·5^a,b,c^	56·1	1a	54·8	1·4^abc^
Amino acid composition (mg/g soft tissue)																				
Aspartic acid	14·1	0·2^a^	13·7	0·1^a^	13·8	0·3^a^	13·9	0·4^a^	14·8	0·2^a^	13·1	0·4^c^	13·4	0·3^b,c^	13·3	0·3^b,c^	14·7	0·3^a,b^	15·7	0·4^a^
Glutamic acid	20·3	0·3^a^	19·7	0·1^a^	19·7	0·5^a^	19·7	0·5^a^	21	0·4^a^	19·2	0·6^b^	19·4	0·3^b^	19·4	0·4^b^	21·2	0·4^a,b^	22·6	0·6^a^
Serine	6·2	0·1^a^	6·6	0^a^	6·4	0·2^a^	5·9	0·2^a^	6·3	0·2^a^	6·4	0·2^a^	6·4	0·2^a^	6·4	0·2^a^	6·6	0·3^a^	7·3	0·3^a^
Histidine	2	0·1^a,b^	1·9	0^b^	1·9	0^a,b^	1·9	0^a,b^	2·1	0^a^	1·9	0·1^a,b^	1·8	0·1^b^	1·8	0^b^	2	0^ab^	2·1	0·1^a^
Glycine	10·7	0·3^a^	10·7	0·1^a^	10·4	0·3^a^	10·8	0·3^a^	11·3	0·3^a^	10·6	0·4^b^	10·5	0·1^b^	10·7	0·4^b^	11·7	0·4^a,b^	13	0·1^a^
Threonine	5·9	0^d,e^	6	0^c,d,e^	6	0^c,d,e^	5·8	0·1^d,e^	6·3	0·1^b,c,d,e^	5·6	0·2^e^	5·7	0·1^e^	5·8	0·1^d,e^	6·2	0·2^b,c,d,e^	6·9	0·3^a,b^
Arginine	12·7	0·1^a^	12·5	0^a^	12·4	0·3^a^	12·5	0·4^a^	13·3	0·3^a^	12·1	0·3^c^	12·2	0·3^b,c^	12·3	0·2^b,c^	13·4	0·3^a,b^	14·3	0·3^a^
Alanine	7·4	0·1^a^	7·3	0·1^a^	7·1	0·2^a^	7·2	0·2^a^	7·6	0·1^a^	7·1	0·2^b^	7·2	0·2^b^	7	0·1^b^	7·8	0·1^a,b^	8·2	0·2^a^
Tyrosine	3·3	0·1^b^	3·4	0^a,b^	3·5	0^a,b^	3·3	0·1^a,b^	3·6	0^a^	3·2	0·2^b^	3·3	0·2^b^	3·4	0·1^a,b^	3·7	0·2^a,b^	4·2	0·2^a^
Valine	6	0·1^a^	5·8	0·1^a^	5·9	0·3^a^	6·1	0·2^a^	6·3	0^a^	6	0·1^a^	5·8	0·1^a^	5·9	0·3^a^	6·1	0·2^a^	6·3	0^a^
Methionine	2·9	0^b,c,d,e^	2·8	0^c,d,e^	2·9	0·1^b,c,d,e^	2·9	0·1^b,c,d,e^	3·1	0·1^a,b,c,d,e^	2·7	0·1^e^	2·7	0^d,e^	2·8	0·1^d,e^	3·2	0·1^a,b,c,d^	3·3	0·1^ab^
Phenylalanine	4·6	0·1^a,b,c,d,e^	4·4	0^c,d,e^	4·5	0·1^c,d,e^	4·5	0·1^b,c,d,e^	4·8	0·1^a,b,c,d^	4·2	0·1^e^	4·3	0·1^d,e^	4·3	0·1^d,e^	4·8	0·1^a,b,c,d^	5·1	0·1^a^
Isoleucine	5·4	0·1^a^	5	0·1^a^	5·1	0·2^a^	5·3	0·2^a^	5·7	0·1^a^	4·7	0·1^b^	4·9	0·1^b^	4·8	0·1^b^	5·6	0·1^a^	5·8	0·1^a^
Leucine	9·1	0·1^a^	8·8	0·1^a^	8·9	0·2^a^	8·9	0·3^a^	9·6	0·2^a^	8·4	0·2^c^	8·6	0·2^b,c^	8·6	0·2^b,c^	9·5	0·2^a,b^	10·2	0·3^a^
Lysine	7·1	0·2	6·7	0·3	7	0·4	6·8	0·5	7·3	0·5	6·5	0·2	6·6	0·3	6·7	0·3	7·2	0·4	7·7	0·9
Proline	7·8	0·1	7·4	0·1	7·5	0·3	7·3	0·2	7·6	0·2	7·6	0·1	7·8	0·1	7·3	0·3	8·1	0·3	8·2	0·2
Total	125·3	1·5^a^	122·9	1^a^	123·1	2·8^a^	122·8	3·7^a^	130·6	2·4^a^	118·8	3·2^b^	120·5	2·1^b^	120·5	2·7^b^	132·1	3·3^ab^	141·3	4^a^

22°C												
P35		P38		P41		P44		P47				
Mean	SEM	Mean	SEM	Mean	SEM	Mean	SEM	Mean	SEM	Protein (A)	Temperature (B)	A x B
736·8	4·3	739·4	1·6	740·2	7·4	745·2	1	741·7	3·5	0·009	*P* < 0·001	0·21
155·3	6·1^b,c,d^	158·4	9·8^b,c,d^	164·6	3·4^a,b,c,d^	165·1	1·4^a,b,c^	175·1	1·8^a^			0·04
18·2	1·2^a,b^	20·5	1·3^a^	21·2	0·4^a^	19·2	0·9^a,b^	20·5	1·2^a^			0·03
19·6	0·2^b,c^	19·8	0·9^a,b,c^	20·4	0·7^a,b,c^	20·2	0·4^a,b,c^	20·3	0·6^a,b,c^			0·002
70·2	2·8^a^	61·9	7·6^a,b^	53·5	9·6^b,c^	50·3	1·3^b,c^	42·4	4·5^c^	*P* < 0·001	*P* < 0·001	0·57
55·9	1·4^a^	56·1	0·8^a^	56·4	1·3^a^	55·2	0·4^a,b^	56·7	0·5^a^			0·05
14·2	0·5^a^	14·1	0·5^a^	14·8	0·6^a^	15·5	0·2^a^	15·8	0·2^a^	*P* < 0·001	*P* < 0·001	0·12
20·9	0·5^a^	20·4	0·8^a^	21·7	1·2^a^	22·2	0·4^a^	22·7	0·5^a^	*P* < 0·001	*P* < 0·001	0·19
6·9	0·3^a^	6·8	0·2^a^	7·2	0·1^a^	7·3	0·3^a^	7·8	0·2^a^	0·011	*P* < 0·001	0·11
2·1	0·1^a^	2	0·1^a^	2·1	0·1^a^	2	0^a^	2·2	0^a^	0·001	*P* < 0·001	0·56
11·6	0·2^a^	11·1	0·6^a^	11·7	0·7^a^	12·4	0·1^a^	13·1	0·2^a^	*P* < 0·001	*P* < 0·001	0·26
6·1	0·2^b,c,d,e^	6·1	0·1^b,c,d,e^	6·6	0·2^a,b,c,d^	6·8	0·2^a,b,c^	7·3	0·1^a^			0·04
13·1	0·3^a^	13·1	0·6^a^	13·4	0·8^a^	13·9	0·2^a^	14·4	0·3^a^	*P* < 0·001	*P* < 0·001	0·33
7·6	0·2^a^	7·3	0·3^a^	7·8	0·4^a^	8	0·1^a^	8·2	0·2^a^	*P* < 0·001	0·002	0·11
3·6	0·1^c^	3·8	0·1^c^	4	0·1^bc^	4·2	0·1^ab^	4·5	0^a^	*P* < 0·001	*P* < 0·001	0·06
5·9	0·1^a^	5·8	0·3^a^	6·1	0·4^a^	6·5	0·1^a^	6·6	0·1^a^	*P* < 0·001	0·16	0·27
2·9	0·1^b,c,d,e^	2·9	0·1^b,c,d,e^	3·3	0·1^a,b,c^	3·2	0·1^a,b,c,d^	3·5	0^a^			0·05
4·5	0·1^b,c,d,e^	4·6	0·1^b,c,d,e^	4·8	0·2^a,b,c,d^	4·9	0^a,b,c^	5·1	0^a,b^			0·04
5·1	0·1^a^	5	0·3^a^	5·3	0·4^a^	5·6	0·1^a^	5·6	0·1^a^	*P* < 0·001	0·34	0·26
9	0·3^a^	9	0·3^a^	9·6	0·5^a^	9·9	0·1^a^	10·3	0·2^a^	*P* < 0·001	0·002	0·12
6·3	0·4	6·8	0·4	6·9	0·8	7·3	0·1	7·8	0·4	0·11	0·97	0·95
7·4	0·3	7·3	0·2	7·6	0·3	7·8	0·2	8	0·2	0·16	0·14	0·2
127·5	3·6^a^	126	4·8^a^	133	7^a^	137·6	1·7^a^	142·8	2·5^a^	*P* < 0·001	0·001	0·18

* See Table 1 for detailed experimental feed information.

† NFE, nitrogen-free extract (calculated).

Values in the same row with different superscripts are significantly different (two-way ANOVA and Tukey’s post hoc test); where there was no significant interaction between the two independent factors, comparisons between protein levels within temperature have been completed (one-way ANOVA and Tukey’s post hoc test).

### Abalone soft tissue amino acid composition

In general, aspartic acid, glutamic acid, glycine and arginine were highly abundant amino acids in abalone tissue irrespective of temperature and dietary protein level (Table 6). At the same time, histidine, tyrosine and methionine were the least abundant amino acids. The vast majority of individual amino acid concentrations were significantly affected by the primary factors of water temperature and dietary protein level, while for the remainder (threonine, methionine and phenylalanine), a significant interaction between these factors was recorded.

In accordance with the total protein levels in the soft tissue of abalone, there was a general trend towards increased individual amino acid concentrations with increasing dietary protein level at each of the water temperatures; however, difference were more evident at the higher water temperature (17 and 22°C). Moreover, the sum of total amino acids was numerically higher in both 17 and 22°C at the higher dietary levels (P44 and P47).

## Discussion

The main objective of the present study was to reveal the optimal protein requirement of farmed Australian hybrid abalone concerning rearing temperature (season). This endeavour was explored to facilitate the timely emergence of hybrid abalone aquaculture in Australia from relative infancy by promoting the efficient growth of farmed abalone during the grow-out phase. Throughout the experiment, abalone showed an active feeding response and there were no mortalities. Furthermore, growth rates were comparable to those on-farm (Jade Tiger Abalone^TM^, pers. comm.) and equal to, or exceeding, the growth rates reported in previous published studies for numerous abalone species^(4,27)^. Ultimately, while differences in growth rates were apparent due to both dietary composition and rearing temperature, which will be discussed further in detail, it was evident that the abalone in the present experiment were cultured within their thermal tolerance limits.

In the present study, there was an observable increase in numerous feed and growth performance parameters as water temperature increased from 12°C to 22°C, including final weight, final shell length, SGR, weight gain percentage and shell growth rate. This concurs with a previous experiment on sub-adult Australian hybrid abalone^(11)^ as well as published studies on other abalone species^(4,12,19,28)^. Several drivers, related to increased metabolic rate and relatedly higher feed consumption^(4,19,28)^, altered digestive physiology and morphology^(29)^, and increased gut enzyme activity^(30,31)^ likely contributed to the differences in growth performance observed.

Given abalone in the current experiment were fed *ad libitum*, the increase in feed consumption with increasing water temperature could be attributed to an increased metabolic requirement^(32)^. In addition, the abalone in the present experiment likely experienced a faster return to appetite at higher water tempeartures given the associated increase in gastric evacuation time^(32,33)^. Therefore, it is evident that within the range of 12–22°C, the feed intake and consequently the growth of hybrid abalone exhibit a positive relationship with water temperature. The understanding of the relationship between water temperature and diet composition and their often interrelated effect on the growth performance of aquaculture species is of fundamental importance to inform on-farm husbandry practices as well as being critical to the development of seasonally specific dietary formulations^(18)^. This is particulary the case in abalone aquaculture, where there is limited control of the environemtnal conditions, inlcuding water temperature, that farmed abalone are subjected to over an entire grow-out period^(11,34)^. In the present experiment, at 12^o^C, dietary protein level significantly affected the final length and shell growth rate where both were higher in abalone fed a diet containing 41 % protein. This trend was observable, although subtle and not significant for other growth performance parameters, including weight gain and SGR, at this lower water temperature. Furthermore, FCR was numerically lower for abalone fed a diet containing 41 % protein, suggesting better feed utilisation. Specifically, an increase in the dietary protein level from 35 % to 41 % yielded a 0·55 g and 16·8 % increase in abalone weight and weight gain percentage, respectively. Therefore, the economic viability of increasing dietary protein level during periods of lower water temperature should be carefully considered owing to the potential cost incursions associated with increasing the concentration of dietary protein in commercial feed formulations. However, as abalone subjected to 12°C experienced lower growth compared with the other temperatures tested, extending the trial duration may have resulted in greater growth disparities between the dietary treatments and therefore made it easier to identify an optimal dietary protein inclusion level during cooler on-farm growing conditions.

Far more definitive results, with respect to differences between the dietary treatments, however, were observed in abalone reared at both 17 and 22°C, where numerous significant differences in feed and growth performance indices were recorded. Specifically, growth performance was significantly improved with an increase in dietary protein level from 35 % to 41 %, before plateauing, and even slightly (although not significantly) declining at the higher dietary protein inclusion levels of 44 and 47 %. Specifically, increasing the dietary protein level beyond 41 % did not improve the final weight, final shell length, SGR, FCR or weight gain percentage. Marked improvements in several growth performance parameters exemplified the performance gains achieved by an increased dietary protein level, including a 74 % increase in weight gain percentage when comparing 35 and 41 % dietary protein in abalone reared at 17°C. A similar improvement was achieved in abalone reared at 22°C where weight gain percentage improved by 66 % as the dietary protein level was increased from 35 % to 41 %. Currently, Australian hybrid abalone are typically fed a diet containing 35 % dietary protein throughout the entire grow-out period based on the nutritional studies performed on Australian greenlip abalone^(3,4,19)^. Clearly, differences exist between the optimal protein requirement for hybrid and greenlip abalone and attest to the importance of investigating the fundamental aspects of aquaculture nutrition, in this case the optimal macronutrient levels, for emerging aquaculture species regardless of perceived similarities to other, more established, species. In addition to the identification of optimal dietary protein level regarding growth performance in hybrid abalone, this study highlighted the temperature-specific (seasonal) nature of maximising potential growth improvements in a farming scenario. It was shown that the implementation of a nutritional strategy, which incorporates a higher dietary protein level (i.e. 41 %), is best applied to periods of faster growth, specifically, where water temperatures exceed 17°C. Such information, if implemented accordingly, is expected to yield considerable production improvements for the Australian hybrid abalone aquaculture industry.

Clearly, modifications to dietary strategies must take into account more than growth performance in order to properly assess the practical feasibility of the proposed alteration^(35)^. For example, excess levels of dietary protein may elicit a series of negative consequences, including a deterioration of the nutritional quality of abalone tissue^(36,37)^, a dietary nutrient imbalance and resultant displacement of other dietary nutrients leading to the catabolism of comparatively expensive dietary protein to meet energy requirements and also negative environmental consequences such as water quality deterioration^(17,21,38,39)^. Further, higher dietary protein inclusion levels may increase the cost of formulated feeds and relatedly the cost of production^(24)^, and this is almost certainly the case for aquafeed formulated for most shellfish species where an increase in dietary protein is typically at the expense of a cheaper raw material (e.g. a carbohydrate source)^(40)^. Therefore, any increase to the dietary protein level, when applied at a commercial level, must be carefully considered in the context of potential performance benefits, feed utilisation and conversion nutrient utilisation and deposition and the nutritional quality of abalone tissue. Therefore, it is important to note that, in the present experiment, FCR was not affected by dietary protein inclusion level across any of the tested temperatures. Higher feed consumption, however, was observed at the higher dietary protein levels at both 17 and 22°C. Furthermore, recorded values for nutrient utilisation indicators (including, protein efficiency ratio, protein deposition, energy efficiency ratio and energy deposition) were higher for abalone fed 41 % dietary protein at both 17 and 22°C, suggesting that both dietary protein and total dietary energy were more efficiently deposited. In addition, the increase in dietary protein level resulted in higher protein and amino acid deposition and reduced nitrogen-free extract content in abalone soft tissue at all three tested temperatures. Considering the concentration of protein and individual amino acids is influential in determining the nutritive value and product quality of shellfish, including abalone^(41,42)^, increasing the dietary protein concentration has the potential to enhance the overall acceptance of the final product delivered to the consumer.

In conclusion, the results revealed in the present study strongly imply that temperature-specific optimisation of protein levels in juvenile Australian hybrid abalone formulated feeds in relation to seasonal water temperature variation could yield significant growth benefits. As a result, inclusion of higher dietary protein levels (41 %) than those currently used commercially (35 %) would significantly improve the growth performance of juvenile Australian hybrid abalone when the water temperature is ≥17°C without any adverse impacts on nutrient utilisation, nutrient deposition or nutritional quality of the abalone soft tissue. Further, it may be beneficial to adhere to the current dietary protein inclusion level (35 %), or a ‘least cost’ feeding approach during cooler seasons, or where water temperatures are ∼12°C, considering the marginal growth improvements that can be achieved during these periods of slow growth.
